# Is it plausible that *Lutzomyia longipalpis* (Psychodidae: Phlebotominae) waited until European colonizers introduced *Leishmania (L.) infantum* to the New World to become a vector of American visceral leishmaniasis? An Opinion

**DOI:** 10.1590/0037-8682-0447-2024

**Published:** 2025-06-02

**Authors:** Fernando Tobias Silveira, Luciana Vieira Lima, Patrícia Karla Ramos, Marliane Batista Campos

**Affiliations:** 1 Instituto Evandro Chagas (Secretaria de Vigilância em Saúde e Meio Ambiente, Ministério da Saúde), Ananindeua, PA, Brasil.; 2 Núcleo de Medicina Tropical, Universidade Federal do Pará, Belém, PA, Brasil.

One of the most contentious issues regarding American visceral leishmaniasis (AVL) is the true origin and taxonomic classification of its etiological agent. The debate over the species *Leishmania chagasi* Cunha & Chagas, 1937 [subsequently revised to *Leishmania (Leishmania) chagasi* Lainson & Shaw, 1987] began in the 1980s. It was then suggested that *Leishmania (L.) infantum* Nicolle, 1908 was actually the causative agent of AVL^1^. This claim was based on comparative enzyme profile analyses at 11 loci involving 110 Brazilian isolates of *L. (L.) chagasi*, sourced from humans, dogs, and *Didelphis* spp. (opossums), which displayed no significant differences from the World Health Organization (WHO) reference strain of *L. (L.) infantum*. In the same period, Grimaldi et al.^2^, using 16 *Leishmania (L.) donovani*-specific monoclonal antibodies, and Kreutzer et al.^3^, analyzing 20 enzyme loci, were also unable to distinguish *L. (L.) chagasi* isolates from Brazil, Honduras, Panama, and Colombia from the WHO reference strain of *L. (L.) infantum*. In the 1990s, Momen et al.^4^ provided additional evidence through enzyme electrophoresis and schizodeme analysis that supported the theory of a recent New World origin for *L. (L.)* chagasi, showing it to be indistinguishable from *L. (L.) infantum*. Towards the decade's end, Mauricio et al.^5^ reported the first findings of genomic diversity within the *L. (L.) donovani* complex and proposed that *L. (L.) chagasi* be recognized as synonymous with *L. (L.) infantum*.

In this century, Maurício et al.^6^ utilized restriction fragment length polymorphism (RFLP) and random amplified polymorphic DNA (RAPD) methods to study this issue. Their findings reinforced the earlier conclusion that *L. (L.) chagasi* is synonymous with *L. (L.) infantum*, suggesting that the latter was likely introduced into the New World primarily by Portuguese and/or Spanish colonizers. Lukes et al.^7^ subsequently conducted a multifactorial genetic analysis, also based on RFLP and RAPD techniques. Their results confirmed that *L. (L.) chagasi* could not be distinguished from *L. (L.) infantum*, leading to the proposal that *L. (L.) donovani* and *L. (L.) infantum* should be the only recognized species within the *L. (L.) donovani* complex.

While acknowledging the taxonomic importance of these molecular/genomic methodologies, it is essential to consider other factors such as clinical, epidemiological, and ecological parameters, including parasite-host interactions and vectors, which are critical for the specific identification of Trypanosomatidae parasites, including *Leishmania* species. It is also important to note that AVL is endemic to a region, Central and South America (Latin America), that exhibits remarkable heterogeneity within the genus *Leishmania*. This region encompasses the three currently recognized subgenera: *L. (Leishmania)* Ross, 1903; *L. (Viannia)* Lainson & Shaw, 1987; and *L. (Mundinia)* Shaw, Camargo & Teixeira, 2016. It includes the highest number of *Leishmania* species (19 in total), with 16 pathogenic to humans [15 causing cutaneous leishmaniasis: *L. (V.) braziliensis*, *L. (V.) peruviana*, *L. (V.) guyanensis*, *L. (V.) panamensis*, *L. (V.) lainsoni*, *L. (V.) shawi*, *L. (V.) naiffi*, *L. (V.) lindenbergi*, *L. (V.) utingensis*, *L. (L.) mexicana*, *L. (L.) amazonensis*, *L. (L.) venezuelensis*, *L. (L.) waltoni*, *L. (L.) ellisi*, *L. (M.) martiniquensis*; and one causing AVL: *L. (L.) chagasi*], and three [*L. (L.) aristidesi*, *L. (L.) garnhami*, and *L. (L.) forattini*] non-pathogenic to humans. Additionally, the origins of these parasites are linked to wild enzootic cycles involving multiple species of sandflies (Psychodidae: Phlebotominae) and a variety of mammals, including rodents, primates, edentates, marsupials, and even felines. This reflects an extraordinary eco-epidemiological factor, underscoring the unique role of Latin American biodiversity in the evolutionary diversity of the genus *Leishmania*, making it the only region globally with representative species across all three recognized subgenera: *L. (Leishmania)*, *L. (Viannia*), and *L. (Mundinia)*.

Based on the premise outlined, the question arises as to why the evolutionary history of the genus *Leishmania* was not more favorable to the Neotropical sandfly *Lutzomyia longipalpis*, which was deprived of interacting with a native New World parasite that could adapt to the microenvironment of its digestive tract. Instead, this interaction occurred only millions of years later with the introduction of *L. (L.) infantum* to the region by European colonizers. It is unlikely that this scenario unfolded as previously speculated. Instead, we have evidence, not merely conjecture, that *L. (L.) chagasi* has an established wild enzootic cycle in the New World, primarily involving its main vector, the sandfly *Lu. longipalpis*. This is supported by the fact that parasites isolated from their wild canid reservoir, the fox *Cerdocyon thous*, in Salvaterra municipality, northern Pará State in Amazonian Brazil^8^, have been dated using molecular clock analysis of the DNA polymerase alpha subunit gene-a highly conserved region in *Leishmania* genomes-to be approximately 143,300 years old, predating *L. (L.) infantum* ^9^.

**FIGURE 1:**
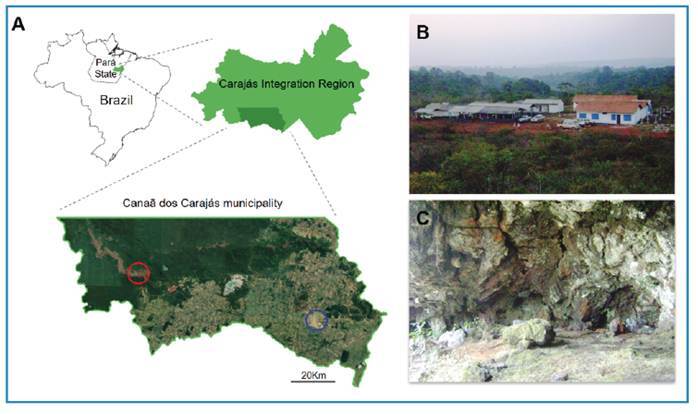
**A:** Geographical placement of Canaã dos Carajás municipality, within the Carajás Integration Region, Pará State, Brazil: blue cycle, urban area of the municipality; red cycle, mineral project area (“Serra Azul”), where biological material (blood and fragments of skin and viscera) from animals (foxes and rodents) and sandflies (*Lutzomyia longipalpis*) were collected; **B:** Mineral enterprise project prospection office; **C:** Speleological cavity; Map processed on QGIS 3.10.7 (https://qgis.org/); vector source: https://www.ibge.gov.br/; raster **source:** https://earth.google.com/.

Furthermore, evidence of the wild enzootic cycle of *L. (L.) chagasi* has been discovered in an isolated forest area within the Canaã dos Carajás municipality, southeast Pará State ('Serra dos Carajás'), part of the iron mineral exploration project 'Serra Sul' by the company 'Vale do Rio Doce'. This area, characterized by restricted human activity and an absence of domestic animals (mainly dogs), hosts *Lu. longipalpis* in caves inhabited by infected *C. thous* foxes (60% seropositive by ELISA using soluble axenic amastigote antigen of the parasite)^10^. Moreover, natural infection by *L. (L.) chagasi* has been confirmed in the viscera (liver and spleen) of the wild rodent *Proechimys* sp. (Rodentia: Echimyidae)^11^ (Figure 1). Additionally, in neighboring Amapá State within the Wajãpi Indigenous Territory-a densely forested area spanning over 12 million hectares-natural infections of *L. (L.) infantum* (*chagasi*) were detected in two wild rodent species, *Dasyprocta* sp. and *Proechimys* cuvieri^12^.

Based on the eco-epidemiological scenario of *Leishmania* parasites in Latin America, the speculative hypothesis suggesting the introduction of *L. (L.) infantum* into the New World lacks ecological plausibility. There is no substantial basis to believe that the infection in domestic animals, brought from Europe, could spread to remote ecotopes of *L. (L.) chagasi* deep within the tropical forests of Amazonian Brazil-areas like the mineral prospecting zone in 'Serra dos Carajás', Pará State, where domestic animals are absent. Furthermore, the notion that such a parasite could transition from a domestic cycle (parasite-vector-dog) to thrive within a wild cycle (parasite-vector-fox or rodent) is unsupported, given that vectors from domestic cycles do not typically revert to wild environments.

In conclusion, while the taxonomic significance of molecular/genomic methodologies in specifically identifying parasites of the genus *Leishmania* is well acknowledged, they should not be considered the definitive tools for determining speciation. In the particular case of *L. (L.) chagasi*, the eco-epidemiological evidence presented here regarding its origin and evolutionary history is compelling and should not be overlooked.
